# The Iron Content of Human Serum Albumin Modulates the Susceptibility of *Acinetobacter baumannii* to Cefiderocol

**DOI:** 10.3390/biomedicines11020639

**Published:** 2023-02-20

**Authors:** Jenny Escalante, Brent Nishimura, Marisel R. Tuttobene, Tomás Subils, Vyanka Mezcord, Luis A. Actis, Marcelo E. Tolmasky, Robert A. Bonomo, María Soledad Ramirez

**Affiliations:** 1 Center for Applied Biotechnology Studies, Department of Biological Science, College of Natural Sciences and Mathematics, California State University Fullerton, Fullerton, CA 92831, USA; 2 Área Biología Molecular, Facultad de Ciencias Bioquímicas y Farmacéuticas, Universidad Nacional de Rosario, Rosario S2000, Argentina; 3 Instituto de Biología Molecular y Celular de Rosario (IBR, CONICET-UNR), Rosario S2000, Argentina; 4 Instituto de Procesos Biotecnológicos y Químicos de Rosario (IPROBYQ, CONICET-UNR), Rosario S2000, Argentina; 5 Department of Microbiology, Miami University, Oxford, OH 45056, USA; 6 Louis Stokes Cleveland Department of Veterans Affairs Medical Center, Research Service and GRECC, Cleveland, OH 44106, USA; 7 Departments of Medicine, Pharmacology, Molecular Biology and Microbiology, Biochemistry, Proteomics and Bioinformatics, Case Western Reserve University School of Medicine, Cleveland, OH 44106, USA; 8 CWRU-Cleveland VAMC Center for Antimicrobial Resistance and Epidemiology (Case VA CARES), Cleveland, OH 44106, USA

**Keywords:** *Acinetobacter baumannii*, human serum albumin, cefiderocol, iron, human pleural fluid, carbapenem-resistance

## Abstract

The mortality rates of patients infected with *Acinetobacter baumannii* who were treated with cefiderocol (CFDC) were not as favorable as those receiving the best available treatment for pulmonary and bloodstream infections. Previous studies showed that the presence of human serum albumin (HSA) or HSA-containing fluids, such as human serum (HS) or human pleural fluid (HPF), in the growth medium is correlated with a decrease in the expression of genes associated with high-affinity siderophore-mediated iron uptake systems. These observations may explain the complexities of the observed clinical performance of CFDC in pulmonary and bloodstream infections, because ferric siderophore transporters enhance the penetration of CFDC into the bacterial cell. The removal of HSA from HS or HPF resulted in a reduction in the minimal inhibitory concentration (MIC) of CFDC. Concomitant with these results, an enhancement in the expression of TonB-dependent transporters known to play a crucial role in transporting iron was observed. In addition to inducing modifications in iron-uptake gene expression, the removal of HSA also decreased the expression of β-lactamases genes. Taken together, these observations suggest that environmental HSA has a role in the expression levels of select *A. baumannii* genes. Furthermore, the removal of iron from HSA had the same effect as the removal of HSA upon the expression of genes associated with iron uptake systems, also suggesting that at least one of the mechanisms by which HSA regulates the expression of certain genes is through acting as an iron source.

## 1. Introduction

The development of novel and effective antibiotic treatments is an urgent need created by the increased number of antibiotic-resistant bacteria. Multi- or pan-drug-resistant *Acinetobacter baumannii* strains, recognized as urgent threats by the Centers for Disease Control and Prevention (CDC), are responsible for serious and often untreatable hospital-acquired infections [1,2]. Especially worrisome are the infections caused by carbapenem-resistant *A. baumannii* (CRAB) strains, for which only a few treatment options are available [3,4,5].

Cefiderocol (CFDC), a recently approved broad-spectrum antibiotic, is one of the few existing options to treat CRAB infections [6,7,8,9,10]. CFDC is a hybrid molecule that consists of a cephalosporin component, which targets cell wall synthesis, linked to a catechol moiety, a chemical addition that facilitates cell penetration by active high affinity ferric siderophore transporters [6,7,8,9,11]. CFDC is approved to treat nosocomial pneumonia and urinary tract infections caused by extensively drug-resistant (XDR) Gram-negative bacteria [4,12,13]. However, the CREDIBLE-CR randomized trial showed that the mortality rates of patients infected with *A. baumannii* and treated with CFDC were higher than the best treatment available for pulmonary and bloodstream infections [4]. In contrast, mortality rates did not increase in CFDC-treated urinary tract infections [4].

Previous work has shown that HSA and unmodified HSA-containing fluids, such as human serum (HS) and human pleural fluid (HPF), modulate the transcriptional expression of various genes associated with several *A. baumannii* functions, including antibiotic resistance, DNA acquisition, and iron uptake [14,15,16,17]. The changes in the transcriptional expression of genes associated with iron uptake systems may be linked to the lower success seen in treating *A. baumannii* infections in humans with CFDC [4,18,19]. The addition of HS, HPF, or purified HSA to the growth medium was associated with an increase in the CFDC MICs of three CRAB-model clinical isolates with different genetic backgrounds [20]. Under these testing conditions, genes that are part of high-affinity iron-uptake systems were downregulated and those associated with resistance to β-lactams were upregulated [20]. In contrast, the addition of human urine (HU), which contains only traces of HSA or free iron, did not result in modifications to CFDC MICs. Moreover, these conditions resulted in an enhancement of the transcription of TonB-dependent receptors (TBDRs) such as *piuA*, *pirA*, *bauA*, and *bfnH* [21]. These results strongly suggested that human bodily fluids with high HSA contents induce changes in the expression of iron uptake and β-lactam resistance-associated genes in *A. baumannii*. It was also observed that modifications to the levels of expression of high-affinity iron uptake system components resulted in variations of the CFDC MIC values [21].

These data lead us to hypothesize that the presence of HSA creates an iron-rich environment that represses the expression of iron-uptake genes, thereby limiting CFDC’s entrance into bacterial cells. In this work, we focus on the analysis of the effect of HSA on CFDC susceptibility and on the expression of genes coding for siderophore-mediated iron acquisition functions and β-lactamases in CRAB. We also show that at least one of the mechanisms by which HSA regulates the transcription of genes coding for siderophore-mediated iron acquisition is associated with HSA acting as an important host iron source that *A. baumannii* encounters during infection.

## 2. Results and Discussion

### 2.1. HSA Present in Human Fluids Alters CFDC MICs via a Global Transcriptional Response

HSA, the predominant protein in human plasma and extracellular fluids, acts as a key host signal triggering an adaptive response in a variety of pathogens and serves as an important host iron reservoir [14,16,20,21,22,23,24,25,26]. For these reasons, we sought to determine whether HSA causes changes in the MIC values of CFDC when *A. baumannii* is exposed to HPF or HS, host fluids that have 50–70% and 90–96% HSA, respectively. For this purpose, the *A. baumannii* CRAB strains AB5075 and AMA40, previously used as model clinical isolates [27,28,29], were cultured in iron-depleted, cation-adjusted Mueller Hinton (CAMHB), 100% HS, or CAMHB supplemented with 4% HPF, as well as in the presence of the cognate HSA-free derivatives of these two human fluids, which were prepared as described in Materials and Methods.

This analysis showed a slight but not significant decrease in the CFDC MIC for strain AB5075 when HSA was removed from both fluids (1 doubling dilution) (Table 1). For strain AMA40, the effect observed was more pronounced when HSA was not present. A decrease of four and seven doubling dilutions was seen when HSA was removed from HS and HPF, respectively (Table 1). Notably, in addition, the appearance of colonies within the growth inhibition zones (heteroresistant cells) was detected in samples cultured in the presence of HS and HPF, but not when bacteria were cultured under HSA depleted conditions.

**Figure 1:**
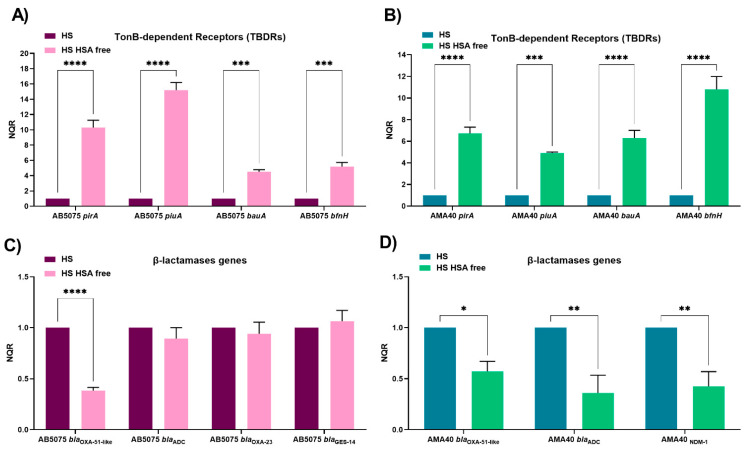
Expression analysis of iron uptake genes and genes coding for β-lactamases in the AB5075 (**A**,**C**) and AMA40 (**B**,**D**) strains. The qRT-PCR of TonB-dependent receptors (*pirA, piuA, bauA, and bfnH*) and *bla* genes (*bla*_OXA-51-like_, *bla*_ADC_, *bla*_OXA-23_, *bla*_NDM-1_, and *bla*_GES-11_) expressed in the presence of human serum (HS) or HSA-free HS. The data shown are the mean ± SD of normalized relative quantities (NRQ) obtained from transcript levels calculated using the qBASE method. This method is a modification of the classic ΔΔCt method used to take multiple reference genes (in this work, *rpoB* and *recA*) and gene-specific amplification efficiencies into account. At least three independent samples were used, and four technical replicates were performed from each sample. The HS condition was used as reference. Statistical significance (*p* < 0.05) was determined by two-way ANOVA followed by Tukey’s multiple comparison test, one asterisk: *p* < 0.05; two asterisks: *p* < 0.01; three asterisks: *p* < 0.001; and four asterisks: *p* < 0.0001.

In order to further verify the role of HSA as a specific inducer of these *A. baumannii* responses, transcriptional analysis via the quantitative RT-PCR (qRT-PCR) of both strains cultured in the presence of HS and HPF and their cognate HSA-free derivatives was also assessed. The expression of the TBDR-dependent *bauA*, *pirA*, *piuA*, and *bfnH* genes, which code for active iron uptake systems, and genes coding for β-lactamases (*bla*_OXA-51-like_, *bla*_ADC_, *bla*_OXA-23_, *bla*_NDM-1_, and *bla*_GES-11_), were evaluated. In both CRAB strains, we observed that the expression of TonB-dependent receptors (TBDRs) genes was significantly increased when HSA was not present in HS (Figure 1A,B, Table S1), suggesting that HSA plays a specific role in triggering the observed changes. As predicted from the consideration of previous observations [21], an opposite result was observed when we analyzed the expression of the β-lactamases genes. A statistically significant decrease in the level of expression of *bla*_OXA-51-like_ was seen when AB5075 was incubated in the presence of HS lacking HSA (Figure 1C, Table S1). Notably, no significant changes were not seen for *bla*_ADC,_ *bla*_OXA-23,_ and *bla*_GES-11_ (Figure 1C, Table S1). For AMA40, statistically significant decreases in transcript levels were seen for the three β-lactamases genes (*bla*_OXA-51-like_, *bla*_ADC_, and *bla*_NDM-1_) present in the strain (Figure 1D, Table S1).

Previous studies have shown a wide transcriptional response (1120 differentially expressed genes) and an impact on CFDC MICs when *A. baumannnii* was exposed to HPF [14,21]. Here, we studied the specific effect of HSA when present in this fluid. Considering that HPF possesses a high HSA-content but also other components, such as reactive oxygen species, monocytes, granulocytes, and other human proteins such the iron chelating protein ferritin, HPF free of HSA was used to assess the differential transcription response of genes coding for TBDRs and β-lactamases.

**Figure 2:**
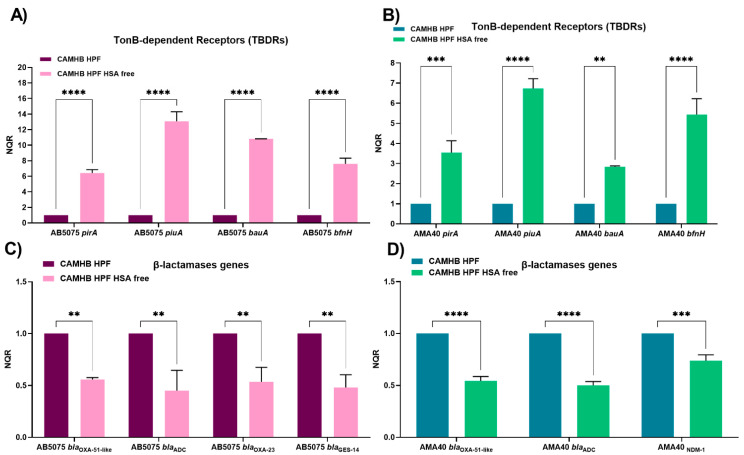
Expression analysis of iron uptake genes and genes coding for β-lactamases in the AB5075 (**A**,**C**) and AMA40 (**B**,**D**) strains. The qRT-PCR of TonB-dependent receptors (*pirA, piuA, bauA, and bfnH*) and *bla* genes (*bla*_OXA-51-like_, *bla*_ADC_, *bla*_OXA-23_, *bla*_NDM-1_, and *bla*_GES-11_) genes expressed in cation-adjusted Mueller Hinton (CAMHB) supplemented with either human pleural fluid (HPF) or HSA-free HPF. The data shown are the means ± SD of normalized relative quantities (NRQ) obtained from transcript levels calculated via the qBASE method. This method is a modification of the classic ΔΔCt method to take multiple reference genes (in this work, *rpoB* and *recA*) and gene-specific amplification efficiencies into account. At least three independent samples were used, and four technical replicates were performed from each sample. The CAMHB HPF was used as reference. Statistical significance (*p* < 0.05) was determined by two-way ANOVA followed by Tukey’s multiple comparison test, two asterisks: *p* < 0.01; three asterisks: *p* < 0.001; and four asterisks: *p* < 0.0001.

Importantly, qRT-PCR results showed that when the *A. baumannii* AB5075 and AMA40 strains were exposed to HSA-free HPF, a statistically significant up-regulation of *bauA*, *pirA*, *piuA*, and *bfnH* occurred when compared with untreated HPF (Figure 2A,B, Table S1). These results, together with those obtained with further HS, support the postulated role HSA plays in regulating the transcriptional expression of *A. baumannii* genes coding for transport functions associated with siderophore-mediated iron acquisition processes.

The transcriptional analysis of the expression of β-lactamases genes in HSA-free HPF showed a statistically significant decrease in the level of expression of all the genes for both *A. baumannii* strains (Figure 2C,D, Table S1).

Taken together, the presented analysis supports the hypothesis that HSA is a significant factor contributing to *A. baumannii* transcriptional responses, which have a major impact on CFDC antibacterial efficacy. In addition, these results further support observations made by Le et al. asserting that fluids with high HSA contents (HPF and HS), or pure HSA at a physiological concentration, down-regulate the expression of iron-uptake systems, while genes associated with β-lactam resistance are up-regulated [29]. In *A. baumannii*, the role of HSA in affecting the expression of genes involved in its antibiotic resistance and pathogenesis has also been previously reported [14,16,17,30,31]. In addition, HSA in combination with carbapenems showed a synergistic increase in natural transformation and expression of competence genes. [29]. Similarly, Ledger et al. showed that HSA directly triggers tolerance to the lipopeptide antibiotic daptomycin in *Staphylococcus aureus* [25]. This tolerance was attributed to the GraRS two-component regulatory system, leading to increased peptidoglycan accumulation as well as another independent mechanism that results in membrane cardiolipin abundance [25]. Notably, these investigators also showed the specific and direct role of HSA as the molecule mediating the observed effects with *S. aureus* [25]. In addition, the role of HSA in augmenting virulence was not only seen in bacteria. In pathogenic fungi, such as *Candida glabrata,* the presence of HSA also contributes to the virulence of this species [26].

### 2.2. Role of Ferric HSA on A. baumannii Response Affecting CFDC Susceptibility

The results obtained with HSA-free fluids strongly support the possibility that HSA is the molecule modulating changes in the expression of genes coding for iron acquisition and β-lactam resistance functions, both of which contribute to alterations in CFDC MICs. However, the mechanisms by which HSA triggers these effects are not yet fully understood. HSA could be playing at least two possible roles: (*i*) HSA exerts a direct role in the modulation of virulence-associated phenotypes; or (*ii)* HSA serves as a carrier of metal ions, such as iron [32,33], affecting the differential expression of genes coding for active iron uptake systems and β-lactam resistance genes. These roles may impact CFDC’s efficacy.

**Figure 3:**
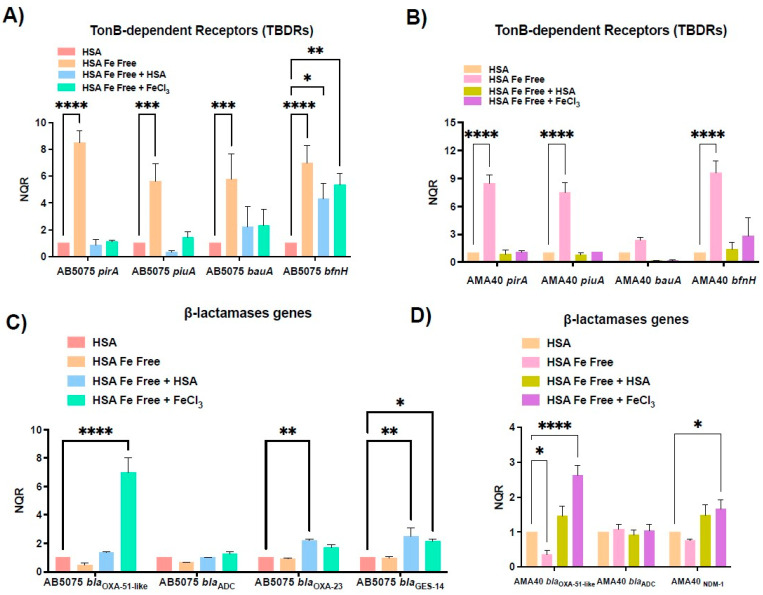
Expression analysis of iron uptake genes and genes coding for β-lactamases in the AB5075 (**A**,**C**) and AMA40 (**B**,**D**) strains. qRT-PCR of *pirA, piuA, bauA, bfnH, bla*_OXA-51-like_, *bla*_ADC_, *bla*_OXA-23_, *bla*_NDM-1_, and *bla*_GES-11_ expressed in human serum albumin (HSA), HSA Fe-free, HSA Fe-free supplemented with FeCl_3_, or HSA Fe-free supplemented with HSA. The data shown are the means ± SD of normalized relative quantities (NRQ) obtained from transcript levels calculated via the qBASE method. This method is a modification of the classic ΔΔCt method used to take multiple reference genes (in this work, *rpoB* and *recA*) and gene-specific amplification efficiencies into account. At least three independent samples were used, and four technical replicates were performed using each sample. The HSA condition was used as a reference. Statistical significance (*p* < 0.05) was determined by two-way ANOVA followed by Tukey’s multiple-comparison test, one asterisk: *p* < 0.05; two asterisks: *p* < 0.01; three asterisks: *p* < 0.001; and four asterisks: *p* < 0.0001.

With the aim of evaluating whether HSA is acting as an iron carrier causing the down-regulation of genes associated with iron-uptake ultimately affecting CFDC activity, iron was removed from HSA (Fe-free HSA). The transcriptional analysis by qRT-PCR showed that when *A. baumannii* was exposed to Fe-free HSA, a statistically significant up-regulation of *bauA*, *pirA*, *piuA*, and *bfnH* occurred in both evaluated strains (Figure 3A,B). In addition, when Fe-free HSA was supplemented with FeCl_3_ or untreated HSA, the transcription expression levels of the tested genes were restored to levels comparable to those of samples incubated with untreated HSA (Figure 3A,B). These results indicate that the iron carried by HSA plays a role in regulating the expression of *A. baumannii* iron-acquisition genes.

Expression levels of β-lactamases genes in Fe-free HSA were next evaluated. *A. baumannii* cells cultured in Fe-free HSA, showed a significant decreased in *bla*_OXA-51-like_ transcripts for AMA40 with respect to the untreated HSA condition (Figure 3D). Significant changes were not seen for either strain in the transcript levels for the other β-lactamases genes under these conditions (Fe-free HSA) (Figure 3C,D). However, when Fe-free HSA was supplemented with FeCl_3_ or HSA, the expression levels were restored (*bla*_OXA-51-like_) or increased compared to those of the HSA condition (Figure 3C,D). Statistically significant increases varied depending on the evaluated gene, condition, and strain (Figure 3C,D).

Studies in vitro have shown that the intrinsic activity of CFDC against *Pseudomonas aeruginosa* is enhanced under iron-limited conditions [9], showing that supplementation with ferric iron increases CFDC MICs. In order to assess whether the same effect occurs in *A. baumannii,* we tested the role of free iron on AMA40 and AB5075 CFDC MICs in vitro. A significant increase in CFDC MICs was noted when the iron-depleted media (CAMHA) was supplemented with 20 µM FeCl_3_ or 40 µM FeCl_3_ (Table S2). These results not only show that the effect of iron on CFDC is independent of the pathogen studied, but also suggest that the variations in the iron content of different human fluids (free iron or iron bound to human proteins) could be a potential factor that affects the efficacy of CFDC.

We next decided to determine the effect of ferric HSA at the phenotypic level through changes in the susceptibility of the bacteria to CFDC. CFDC MICs for AMA40 and AB5075 using CAMHB (untreated), CAMHB supplemented with HSA pre-Chelex^®^ treatment (HSA Fe), or Fe-Free HSA were performed. In addition, the CAMHB was supplemented with Fe-Free HSA + 100 µM FeCl_3_ and Fe-Free HSA + 3.5% HSA in order to further determine the role in CFDC susceptibility. The minimal bactericidal concentration (MBC) was also determined under these conditions while keeping in mind the occurrence of heteroresistance that cannot be detected using the microdilution method. A decrease was observed in the MIC and MBC values for AB5075 when the iron was removed from HSA; values were restored or even further increased more when inorganic iron was added back to the Fe-free HSA tested condition (Table 2). A similar response was observed when untreated HSA was added to the medium. Similarly, in the AMA40 strain, MIC and MBC values decreased when iron was removed, while restored or increased when iron or HSA were added (Table 2). These results indicate that iron-rich HSA and/or the presence of free inorganic iron are associated with reduced susceptibility to CFDC.

### 2.3. Changes in the Expression of Genes Coding for Iron Uptake Functions and β-Lactam Resistance in Cerebrospinal Fluid (CSF), a Low HSA Content Fluid

Previous work showed that a human fluid, such as urine, with no or a trace amount of HSA triggers different *A. baumannii* behavioral and transcriptional responses [21]. This work also showed that CFDC MICs values are not significantly modified, while the expression of *piuA*, *pirA*, *bauA*, and *bfnh* was enhanced when bacteria were cultured in urine, suggesting that CFDC uptake through active iron transport systems is not impaired [21].

**Figure 4:**
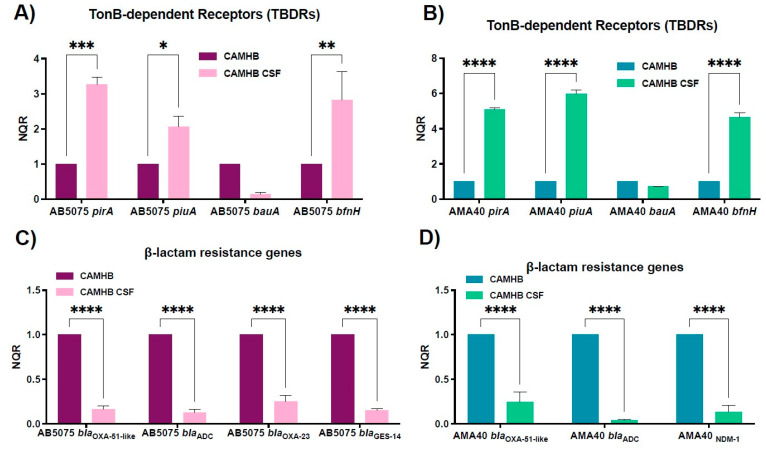
Expression analysis of iron uptake genes and genes coding for β-lactamases in the AB5075 (**A**,**C**) and AMA40 (**B**,**D**) strains. qRT-PCR of TonB-dependent receptors *(pirA, piuA, bauA,* and *bfnH)* and *bla* genes (*bla*_OXA-51-like_, *bla*_ADC_, *bla*_OXA-23_, *bla*_NDM-1_, and *bla*_GES-11_) expressed in cation-adjusted Mueller Hinton (CAMHB) or CAMHB supplemented with cerebrospinal fluid (CSF). The data shown are the means ± SD of normalized relative quantities (NRQ) obtained from transcript levels calculated by the qBASE method. This method is a modification of the classic ΔΔCt method used to take multiple reference genes (in this work, *rpoB* and *recA*) and gene-specific amplification efficiencies into account. At least three independent samples were used. CAMHB was used as the reference condition. Statistical significance (*p* < 0.05) was determined by two-way ANOVA followed by Tukey’s multiple-comparison test, one asterisk: *p* < 0.05; two asterisks: *p* < 0.01; three asterisks: *p* < 0.001; and four asterisks: *p* < 0.0001.

Since the incidence of *A. baumannii* as a causal agent of nosocomial meningitis has been increasing in recent years with a mortality rate of 15–71% [34,35], and knowing that only 1% of the cerebrospinal fluid (CSF) content corresponds to proteins, HSA representing 70% of these proteins [36], we decided to study the expression of genes coding for iron uptake systems and β-lactams resistance when *A. baumannii* cells are exposed to 20% CSF. It is important to note that our analysis resulted in no detectable iron in 100% and 20% CSF samples (see Table S3). qRT-PCR showed that the exposure of *A. baumannii* to CSF resulted in a statistically significant up-regulation of *piuA*, *pirA*, and *bfnH* with both tested strains when compared to the CAMHB control (Figure 4A,B). In contrast, *bauA* transcription was down-regulated, although not significantly, when compared to the same control (Figure 4A,B). These observations agree with the results of Nishimura et al., showing a similar differential expression of iron acquisition genes when *A. baumannii* encounters human fluids with low HSA contents [21]. Moreover, the expression levels of β-lactamases genes are also affected by the presence of CSF; promoting the statistically significant down-regulation of *bla* genes tested in both strains (Figure 4C,D). As expected, considering the previous results seen in urine, changes in the MIC to CFDC were not observed when *A. baumannii* AB5075 or AMA40 were exposed to CSF (Table S3).

In summary, these results support the hypothesis that the absence of detectable iron and the low HSA content of CSF compared to HS or HPF are the signals that affect the antimicrobial activity of CFDC.

## 3. Concluding Remarks

The results obtained in the present work further expand upon previous investigations and provide insights into earlier observations suggesting that human fluids containing high concentrations of HSA, including HPF and HS, affect the potency of CFDC as an antibacterial agent against *A. baumannii*. Here, we demonstrate that HSA is a critical host product that significantly affects the antimicrobial efficacy of CFDC. This undesirable outcome is due to the capacity of HSA and HSA-containing fluids to act as a viable iron source, which ultimately downregulates the transcriptional expression of genes that code for the active transport of *A. baumannii* high-affinity siderophores, particularly those that include a catechol moiety. Our data show that such a response correlates with significant increases in CFDC MICs. On the other hand, the mechanism by which the presence of ferric HSA controls the differential expression of genes coding for β-lactam resistance remains to be elucidated.

It must be noted that this study was conducted on two *A. baumannii* strains. Considering the high genetic variability of this bacterium, we cannot be sure that the phenomenon is present in all strains of *A. baumannii*. Also, this investigation was conducted using pure fluids (a highly controlled environment). In contrast, the milieu wherein humans suffer from infections is very complex. We know that *A. baumannii* has numerous clinical manifestations and can cause different disease states. In each of these cases there may be multiple effects on gene expression. It would therefore be premature to extrapolate our in vitro results to what happens in the human body. Experiments using animal models of infection will help to assess the clinical environments’ impact on *A. baumannii*, regulating gene expression to modify levels of resistance to CFDC.

## 4. Materials and Methods

### 4.1. Bacterial Strains

The carbapenem-resistant *A. baumannii* AB5075 (*bla*_OXA-51-like_, *bla*_ADC_, *bla*_OXA-23_, and *bla*_GES-11_) [27,29,31] model strain and the clinical carbapenem-resistant AMA40 (*bla*_OXA-51-like_, *bla*_ADC,_ and *bla*_NDM-1_) isolate [21,28,37], belonging to different clonal complexes, were used in this work.

### 4.2. RNA Extraction, Quantitative Reverse Transcription Polymerase Chain Reaction (qRT-PCR)

*A. baumannii* AB5075 and AMA40 cells were cultured in iron depleted cation adjusted Mueller Hinton (CAMHB) and incubated with agitation for 18 h at 37 °C. Overnight cultures were then diluted 1:10 in fresh CAMHB, CAMHB supplemented with 3.5% human serum albumin (HSA), 3.5% iron-free HSA, 3.5 iron-free HSA + 100 µM FeCl_3_, 3.5% iron-free HSA + 100 µM FeCl_3_ + 3.5% HSA. Bacteria were also cultured in CAMHB supplemented with 4% human pleural fluid (HPF), CAMHB supplemented with HSA-free HPF, 100% human serum (HS) or 100% HSA free HS. All samples were incubated with agitation for 18 h at 37 °C. RNA was immediately extracted using the Direct-zol RNA Kit (Zymo Research, Irvine, CA, USA) following the manufacturer’s instructions, as previously described [21]. Total RNA extractions were performed in three biological replicates for each condition. The extracted and DNase-treated RNA was used to synthesize cDNA iScriptTM Reverse Transcription Supermix for qPCR (Bio-Rad, Hercules, CA, USA) using the manufacturer’s protocol. The cDNA concentrations were adjusted to 50 ng/µL, and qPCR was conducted using the qPCRBIO SyGreen Blue Mix Lo-ROX following the manufacturer’s protocol (PCR Biosystems, Wayne, PA, USA). At least three biological replicates of cDNA were each tested in triplicate using the CFX96 TouchTM Real-Time PCR Detection System (Bio-Rad, Hercules, CA, USA). Data are presented as NRQ (normalized relative quantities) calculated by the qBASE method [38], using *recA* and *rpoB* genes as normalizers. The qBASE method is a modification of the classic ΔΔCt method used to take multiple reference genes and gene-specific amplification efficiencies into account [38]. The sequences of the qPCR primers are listed in Table S4. Asterisks indicate significant differences as determined by *t*-test or ANOVA followed by Tukey’s multiple comparison test (*p* < 0.05), using GraphPad Prism (GraphPad software, San Diego, CA, USA).

### 4.3. HSA Removal

In order to remove the HSA from HS or HPF, the ProteoExtract^®^ Albumin/IgG Removal Kit (Sigma-Aldrich, St. Louis, MO, USA) was used following the manufacturer’s instructions. To corroborate the correct removal of HSA, protein samples of both fluids pre- and post HSA removal treatment were separated by linear SDS-PAGE (REF) (12% resolving and 6% stacking gel) and visualized by Coomassie staining (Figure S1). Protein concentrations in all of the soluble extracts analyzed (Table S5) were determined by the Bradford method [39].

### 4.4. Iron Removal

Iron was removed from HSA samples using Chelex^®^ 100 Chelating Ion Exchange Resin (Bio-Rad, Hercules, CA, USA) following the manufacturer’s instructions. The iron content of pre- and post Chelex^®^ 100 treatment was determined using the Iron Assay Kit (Sigma-Aldrich, St. Louis, MA, USA) following the manufacturer’s recommendations (Table S6).

### 4.5. Antimicrobial Susceptibility Testing

Antibiotic susceptibility assays were performed following the procedures recommended by the Clinical and Laboratory Standards Institute (CLSI). After OD_600_ adjustment, 100 µL of *A. baumannii* AB5075 and AMA40 cells grown in fresh CAMHB, or CAMHB supplemented with human fluids (HPF, HS, or CSF) or fluids where HSA was removed (HSA-free HPF and HSA-free HS) were inoculated on CAMH agar plates (CAMHA) as previously described [21]. Antimicrobial commercial E-strips (Liofilchem S.r.l., Roseto degli Abruzzi, Italy) CFDC were used. CAMHA plates were incubated at 37 °C for 18 h. CLSI breakpoints were used for interpretation [40]. *E. coli* ATCC 25922 was used for quality control purposes.

The microdilution test was used to study the effect of HSA and iron-free HSA on CFDC MICs. CAMHB was prepared as described above and supplemented with HSA, iron-free HSA, iron-free HSA Fe + 100 µM FeCl_3_, or iron-free HSA + iron-free HSA Fe + 3.5% HSA to performed CFDC (range 0.25–512 mg/L) following the CLSI guidelines [40]. In order to study the role of ferric iron on AMA40 and AB5075 CFDC MIC, iron-depleted CAMHA and iron-depleted CAMHA supplemented with 20 µM FeCl_3_ or 40 µM FeCl_3_ were used. The MICs were determined using CFDC MTS strips (Liofilchem S.r.l., Italy) following the CLSI guidelines [40]. The quality control strain (*Escherichia coli* ATCC 25922) was used as a control [40,41]. CLSI breakpoints were used for interpretation [40].

## Figures and Tables

**Table 1:** Minimal inhibitory concentrations (MICs) of cefiderocol (CFDC) for the CRAB AB5075 and AMA40 strains, calculated using CFDC MTS strips (Liofilchem S.r.l., Italy) on iron-depleted CAMHA (cation-adjusted Mueller Hinton agar) and the different conditions tested.

Condition	CFDC MIC (mg/L)	
	AB5075	AMA40
CAMHB	0.5 (S)	0.5 (S)
4% HPF	1 (S)	16 * (R)
4% HPF HSA free **	0.5 (S)	0.25 (S)
100% HS	1 (S)	4 * (S)
100% HS HSA free **	0.5 (S)	0.25 (S)

CFDC: cefiderocol, S: susceptible, R: resistant; * intra-colonies are present. ** HSA removal, Sigma Aldrich.

**Table 2:** The minimal inhibitory concentrations (MICs) and minimal bactericidal concentrations (MBCs) of CFDC for the CRAB AB5075 and AMA40 strains calculated via microdilution on iron-depleted CAMHB and the different conditions tested.

Strains	CFDC			
	AB5075		AMA40	
	MIC (mg/L)	MBC (mg/L)	MIC (mg/L)	MBC (mg/L)
Untreated	0.25 (S)	0.25 (S)	0.5 (S)	32 (R)
HSA pre-Chelex^®^ treatment	8 (I)	32 (R)	2 (S)	64 (R)
HSA Fe-Free (post-Chelex^®^ treatment)	0.125 (S)	8 (I)	1 (S)	16 (R)
HSA Fe-Free + 100µM FeCl_3_	32 (R)	256 (R)	128 (R)	128 (R)
HSA Fe-Free + 3.5% HSA	8 (I)	64 (R)	4 (S)	64 (R)

CFDC: cefiderocol, S: susceptible, I: intermediate, R: resistant.

## Supplementary Materials

The following supporting information can be downloaded at: https://www.mdpi.com/article/10.3390/biomedicines11020639/s1, Table S1: Comparison of the level of expression of iron associated and antibiotic resistance genes obtained by qRT-PCR in the two CRAB strains, Table S2: Minimal Inhibitory Concentrations (MICs) of cefiderocol (CFDC) for the CRAB AB5075 and AMA40 strains performed using CFDC MTS strips (Liofilchem S.r.l., Italy) on Iron-depleted CAMHA (Cation Adjusted Mueller Hinton Agar) supplemented with 20 µM or 40 µM FeCl3, Table S3: Minimal Inhibitory Concentrations (MICs) of cefiderocol (CFDC) for the CRAB AB5075 and AMA40 strains, performed using CFDC MTS strips (Liofilchem S.r.l., Italy) on Iron-depleted CAMHA (Cation Adjusted Mueller Hinton Agar) and supplemented with 4% or 20% of cerebrospinal fluid (CSF), Table S4. Name of the primers used in the present study and their corresponding sequences, Table S5. Total protein concentration determined in CAMHB, and different fluids analyzed using the ProteoExtract® Albumin/IgG Removal Kit (Sigma-Aldrich, St. Louis, MA, USA), Table S6. Total Iron concentration determined in CAMHB, and different fluids analyzed using the Iron Assay Kit (Sigma-Aldrich, St. Louis, MA, USA), Figure S1. SDS-PAGE of human fluids analyzed using the ProteoExtract® Albumin/IgG Removal Kit (Sigma-Aldrich, St. Louis, MA, USA).

## References

[1] CDC, US Department of Health and Human Services, Centres for Disease Control and Prevention (2019). Antibiotic Resistance Threats in the United States.

[2] Isler B., Doi Y., Bonomo R.A., Paterson D.L. (2019). New treatment options against carbapenem-resistant *Acinetobacter baumannii* infections. Antimicrob. Agents Chemother..

[3] Karaiskos I., Lagou S., Pontikis K., Rapti V., Poulakou G. (2019). The “Old” and the “New” Antibiotics for MDR Gram-Negative Pathogens: For Whom, When, and How. Front. Public Health.

[4] Bassetti M., Echols R., Matsunaga Y., Ariyasu M., Doi Y., Ferrer R., Lodise T.P., Naas T., Niki Y., Paterson D.L. (2021). Efficacy and safety of cefiderocol or best available therapy for the treatment of serious infections caused by carbapenem-resistant Gram-negative bacteria (CREDIBLE-CR): A randomised, open-label, multicentre, pathogen-focused, descriptive, phase 3 trial. Lancet Infect. Dis..

[5] Bassetti M., Peghin M., Vena A., Giacobbe D.R. (2019). Treatment of Infections Due to MDR Gram-Negative Bacteria. Front. Med..

[6] Bonomo R.A. (2019). Cefiderocol: A Novel Siderophore Cephalosporin Defeating Carbapenem-resistant Pathogens. Clin. Infect. Dis..

[7] Zhanel G.G., Golden A.R., Zelenitsky S., Wiebe K., Lawrence C.K., Adam H.J., Idowu T., Domalaon R., Schweizer F., Zhanel M.A. (2019). Cefiderocol: A Siderophore Cephalosporin with Activity Against Carbapenem-Resistant and Multidrug-Resistant Gram-Negative Bacilli. Drugs.

[8] Aoki T., Yoshizawa H., Yamawaki K., Yokoo K., Sato J., Hisakawa S., Hasegawa Y., Kusano H., Sano M., Sugimoto H. (2018). Cefiderocol (S-649266), A new siderophore cephalosporin exhibiting potent activities against *Pseudomonas aeruginosa* and other gram-negative pathogens including multi-drug resistant bacteria: Structure activity relationship. Eur. J. Med. Chem..

[9] Ito A., Nishikawa T., Matsumoto S., Yoshizawa H., Sato T., Nakamura R., Tsuji M., Yamano Y. (2016). Siderophore Cephalosporin Cefiderocol Utilizes Ferric Iron Transporter Systems for Antibacterial Activity against Pseudomonas aeruginosa. Antimicrob. Agents Chemother..

[10] McCreary E.K., Heil E.L., Tamma P.D. (2021). New Perspectives on Antimicrobial Agents: Cefiderocol. Antimicrob. Agents Chemother..

[11] Parsels K.A., Mastro K.A., Steele J.M., Thomas S.J., Kufel W.D. (2021). Cefiderocol: A novel siderophore cephalosporin for multidrug-resistant Gram-negative bacterial infections. J. Antimicrob. Chemother..

[12] Wunderink R.G., Matsunaga Y., Ariyasu M., Clevenbergh P., Echols R., Kaye K.S., Kollef M., Menon A., Pogue J.M., Shorr A.F. (2021). Cefiderocol versus high-dose, extended-infusion meropenem for the treatment of Gram-negative nosocomial pneumonia (APEKS-NP): A randomised, double-blind, phase 3, non-inferiority trial. Lancet Infect. Dis..

[13] Jacobs M.R., Abdelhamed A.M., Good C.E., Rhoads D.D., Hujer K.M., Hujer A.M., Domitrovic T.N., Rudin S.D., Richter S.S., van Duin D. (2019). ARGONAUT-I: Activity of Cefiderocol (S-649266), a Siderophore Cephalosporin, against Gram-Negative Bacteria, Including Carbapenem-Resistant Nonfermenters and *Enterobacteriaceae* with Defined Extended-Spectrum beta-Lactamases and Carbapenemases. Antimicrob. Agents Chemother..

[14] Martinez J., Fernandez J.S., Liu C., Hoard A., Mendoza A., Nakanouchi J., Rodman N., Courville R., Tuttobene M.R., Lopez C. (2019). Human pleural fluid triggers global changes in the transcriptional landscape of *Acinetobacter baumannii* as an adaptive response to stress. Sci. Rep..

[15] Martinez J., Razo-Gutierrez C., Le C., Courville R., Pimentel C., Liu C., Fung S.E., Tuttobene M.R., Phan K., Vila A.J. (2021). Cerebrospinal fluid (CSF) augments metabolism and virulence expression factors in *Acinetobacter baumannii*. Sci. Rep..

[16] Quinn B., Rodman N., Jara E., Fernandez J.S., Martinez J., Traglia G.M., Montana S., Cantera V., Place K., Bonomo R.A. (2018). Human serum albumin alters specific genes that can play a role in survival and persistence in *Acinetobacter baumannii*. Sci. Rep..

[17] Rodman N., Martinez J., Fung S., Nakanouchi J., Myers A.L., Harris C.M., Dang E., Fernandez J.S., Liu C., Mendoza A.M. (2019). Human Pleural Fluid Elicits Pyruvate and Phenylalanine Metabolism in *Acinetobacter baumannii* to Enhance Cytotoxicity and Immune Evasion. Front. Microbiol..

[18] Gatti M., Bartoletti M., Cojutti P.G., Gaibani P., Conti M., Giannella M., Viale P., Pea F. (2021). A descriptive case series of pharmacokinetic/pharmacodynamic target attainment and microbiological outcome in critically ill patients with documented severe extensively drug-resistant *Acinetobacter baumannii* bloodstream infection and/or ventilator-associated pneumonia treated with cefiderocol. J. Glob. Antimicrob. Resist..

[19] Falcone M., Tiseo G., Leonildi A., Della Sala L., Vecchione A., Barnini S., Farcomeni A., Menichetti F. (2022). Cefiderocol- Compared to Colistin-Based Regimens for the Treatment of Severe Infections Caused by Carbapenem-Resistant *Acinetobacter baumannii*. Antimicrob. Agents Chemother..

[20] Le C., Pimentel C., Pasteran F., Tuttobene M.R., Subils T., Escalante J., Nishimura B., Arriaga S., Carranza A., Mezcord V. (2022). Human Serum Proteins and Susceptibility of *Acinetobacter baumannii* to Cefiderocol: Role of Iron Transport. Biomedicines.

[21] Nishimura B., Escalante J., Tuttobene M.R., Subils T., Mezcord V., Pimentel C., Georgeos N., Pasteran F., Rodriguez C., Sieira R. (2022). *Acinetobacter baumannii* response to cefiderocol challenge in human urine. Sci. Rep..

[22] Pinsky M., Roy U., Moshe S., Weissman Z., Kornitzer D. (2020). Human Serum Albumin Facilitates Heme-Iron Utilization by Fungi. mBio.

[23] Egesten A., Frick I.M., Morgelin M., Olin A.I., Bjorck L. (2011). Binding of albumin promotes bacterial survival at the epithelial surface. J. Biol. Chem..

[24] Gonyar L.A., Gray M.C., Christianson G.J., Mehrad B., Hewlett E.L. (2017). Albumin, in the Presence of Calcium, Elicits a Massive Increase in Extracellular Bordetella Adenylate Cyclase Toxin. Infect. Immun..

[25] Ledger E.K., Mesnage S., Edwards A.M. (2022). Human serum triggers antibiotic tolerance in *Staphylococcus aureus*. Nat. Commun..

[26] Pekmezovic M., Kaune A.K., Austermeier S., Hitzler S.U.J., Mogavero S., Hovhannisyan H., Gabaldón T., Gresnigt M.S., Hube B. (2021). Human albumin enhances the pathogenic potential of Candida glabrata on vaginal epithelial cells. PLoS Pathog.

[27] Jacobs A.C., Thompson M.G., Black C.C., Kessler J.L., Clark L.P., McQueary C.N., Gancz H.Y., Corey B.W., Moon J.K., Si Y. (2014). AB5075, a Highly Virulent Isolate of *Acinetobacter baumannii*, as a Model Strain for the Evaluation of Pathogenesis and Antimicrobial Treatments. MBio.

[28] Rodgers D., Pasteran F., Calderon M., Jaber S., Traglia G.M., Albornoz E., Corso A., Vila A.J., Bonomo R.A., Adams M.D. (2020). Characterisation of ST25 NDM-1-producing *Acinetobacter* spp. strains leading the increase in NDM-1 emergence in Argentina. J. Glob. Antimicrob. Resist..

[29] Le C., Pimentel C., Tuttobene M.R., Subils T., Nishimura B., Traglia G.M., Perez F., Papp-Wallace K.M., Bonomo R.A., Tolmasky M.E. (2021). Interplay between meropenem and human serum albumin on expression of carbapenem resistance genes and natural competence in *Acinetobacter baumannii*. Antimicrob. Agents Chemother..

[30] Martinez J., Liu C., Rodman N., Fernandez J.S., Barberis C., Sieira R., Perez F., Bonomo R.A., Ramirez M.S. (2018). Human fluids alter DNA-acquisition in *Acinetobacter baumannii*. Diagn Microbiol. Infect. Dis..

[31] Pimentel C., Le C., Tuttobene M.R., Subils T., Martinez J., Sieira R., Papp-Wallace K.M., Keppetipola N., Bonomo R.A., Actis L.A. (2021). Human Pleural Fluid and Human Serum Albumin Modulate the Behavior of a Hypervirulent and Multidrug-Resistant (MDR) *Acinetobacter baumannii* Representative Strain. Pathogens.

[32] Bal W., Sokolowska M., Kurowska E., Faller P. (2013). Binding of transition metal ions to albumin: Sites, affinities and rates. Biochim. Biophys Acta.

[33] Merlot A.M., Kalinowski D.S., Richardson D.R. (2014). Unraveling the mysteries of serum albumin-more than just a serum protein. Front. Physiol..

[34] Kim B.N., Peleg A.Y., Lodise T.P., Lipman J., Li J., Nation R., Paterson D.L. (2009). Management of meningitis due to antibiotic-resistant *Acinetobacter* species. Lancet Infect. Dis..

[35] Xiao J., Zhang C., Ye S. (2019). *Acinetobacter baumannii* meningitis in children: A case series and literature review. Infection.

[36] Ramström M., Zuberovic A., Grönwall C., Hanrieder J., Bergquist J., Hober S. (2009). Development of affinity columns for the removal of high-abundance proteins in cerebrospinal fluid. Biotechnol. Appl. Biochem..

[37] Adams M.D., Pasteran F., Traglia G.M., Martinez J., Huang F., Liu C., Fernandez J.S., Lopez C., Gonzalez L.J., Albornoz E. (2020). Distinct mechanisms of dissemination of NDM-1 metallo- beta-lactamase in *Acinetobacter* spp. in Argentina. Antimicrob. Agents Chemother..

[38] Hellemans J., Mortier G., De Paepe A., Speleman F., Vandesompele J. (2007). qBase relative quantification framework and software for management and automated analysis of real-time quantitative PCR data. Genome Biol..

[39] Bradford M.M. (1976). A rapid and sensitive method for the quantitation of microgram quantities of protein utilizing the principle of protein-dye binding. Anal. Biochem..

[40] (2020). Performance Standards for Antimicrobial Susceptibility Testing: Thirty Edition Informational Supplement.

[41] Huband M.D., Ito A., Tsuji M., Sader H.S., Fedler K.A., Flamm R.K. (2017). Cefiderocol MIC quality control ranges in iron-depleted cation-adjusted Mueller-Hinton broth using a CLSI M23-A4 multi-laboratory study design. Diagn Microbiol. Infect. Dis..

