# Supplementary material for: The Iron Content of Human Serum Albumin Modulates the Susceptibility of Acinetobacter baumannii to Cefiderocol
Source: Biomedicines. 2023 Feb 20;11(2):639. doi: 10.3390/biomedicines11020639 (PMC9953112; doi:10.3390/biomedicines11020639)
Supplement: Supplementary file 1 [file biomedicines-11-00639-s001.zip › biomedicines-2132851-supplementary.pdf]

Table S1: Comparison of the level of expression of iron associated and antibiotic resistance genes obtained by qRT-PCR in the two CRAB strains

Gene name	AB5075 (Log_2FC)		AMA40 (Log_2FC)	
	HS HSA free	HPF HSA free	HS HSA free	HPF HSA free
TonB-dependent Receptors (TBDRs)				
pirA	3.36	2.68	2.75	1.82
piuA	3.92	3.70	2.29	2.75
bauA	2.18	3.44	2.65	1.50
bfnH	2.37	2.92	3.43	2.43
β-lactamases genes				
bla$_{OXA-51}$	-1.38	-0.84	-0.82	-0.88
bla$_{ADC}$	-0.16	-1.14	-1.47	-1
bla$_{OXA-23}$	-0.09	-0.89		
bla$_{GES-14}$	0.08	-1.05		
bla$_{NDM-1}$			-1.25	-0.44

$Log_2FC > 1$ ($p<0.05$)
$Log_2FC < -1$ ($p<0.05$)
Log_2FC (0-1)
Log_2FC (-1-0)

Table S2. CFDC MICs for two CRAB representative strains performed using CFDC MTS strips (Liofilchem S.r.l., Italy) on Iron-depleted CAMHA (Cation Adjusted Mueller Hinton Agar) supplemented with 20 µM or 40 µM $FeCl_3$.

Strain	CAMHA	CAMHA + 20 µM $FeCl_3$	CAMHA + 40 µM $FeCl_3$
AMA40	0.25	1.5	3.0
AB5075	0.19	1.5	2.0

CAMHA: Iron-depleted Cation Adjusted Mueller Hinton (treated with CHELEX as recommend by the CLSI).

Table S3: Minimal Inhibitory Concentrations (MICs) of cefiderocol (CFDC) for the CRAB AB5075 and AMA40 strains, performed using CFDC MTS strips (Liofilchem S.r.l., Italy) on Iron-depleted CAMHA (Cation Adjusted Mueller Hinton Agar) and supplemented with 20% of cerebrospinal fluid (CSF).

Condition	CFDC MIC (*mg*/L)	
	AB5075	AMA40
CAMHB	0.50 (S)	0.50 (S)
20% CSF	0.38 (S)	0.75 (S)

CFDC: cefiderocol, S: Susceptible, R: Resistant

Table S4. Name of the primers used in the present study and their corresponding sequences.

Primer Name	Function	5'-3' Sequence
bla$_{OXA-51-like}$ F	β-lactamase	ATGAACATTAAAACACTCTT
bla$_{OXA-51-like}$ R	β-lactamase	TATAAAATACCTAATTGTTC
bla$_{OXA-23}$ F	β-lactamase	CGAGTCAGATTGTTCAAGGA
bla$_{OXA-23}$ R	β-lactamase	GCTCAACCCAGCCGGTCAAC
bla$_{GES-14}$ F	β-lactamase	ACGCACTATTACTGGCAGGG
bla$_{GES-14}$ R	β-lactamase	TCTCCTTGGGGATCGACGAT
bla$_{NDM-1}$ F	β-lactamase	GAAGCTGAGCACCGCATTAG
bla$_{NDM-1}$ R	β-lactamase	AACCAGATCGCCAAACCGTT
bauA F	Iron uptake	AAATGTTTGGCCGCGTTGAGGT
bauA R	Iron uptake	CAATCGTGCAAACGGTTCATCAGC
pirA F	Iron uptake	GTCTATGGCTTTTGCTGCACA
pirA R	Iron uptake	GCGATTGCTTCACTTGCTCT
piuA F	Iron uptake	ATGGCGCAAGAAGCAGTTTC
piuA R	Iron uptake	TGTCTTTGAGAGGAGCCACG
bfnH F	Iron uptake	ACTGCGACTCGTACACCAAA
bfnH R	Iron uptake	ACTTTACGACCTGCCGTAGC
bla$_{ADC}$ F *(ampC)*	β-lactamase	TGCCAACCTTAACCCACA
bla$_{ADC}$ R *(ampC)*	β-lactamase	AGTTTGTAACGTTGCCGG

Table S5. Total protein concentration determined in CAMHB and different fluids analyzed using the ProteoExtract® Albumin/IgG Removal Kit (Sigma-Aldrich, MA, United States) following the manufacturer's recommendations.

	Protein concentration (mg/mL)	Reference
CAMHB	BLQ	This work
HPF	22.66 ± 4.53	This work
HPF HSA Free	BLQ	This work
HS	14.15 ± 0.31	This work
HS HSA Free	BLQ	This work

CAMHB: iron depleted cation adjusted Mueller Hinton, HSA: human serum albumin, HPF: human pleural fluids, HS: human serum, BLQ: below limit of quantification.

Table S6. Total Iron concentration determined in CAMHB and different fluids analyzed using the Iron Assay Kit (Sigma-Aldrich, MA, United States) following the manufacturer's recommendations.

	[Fe] total (µM)	Reference
CAMHB	BLQ	This work
HSA	65.4 ± 0.39	This work
HSA Fe Free	BLQ	This work
HPF	129.97 ± 0.86	[1]
HPF HSA Free	BLQ	This work
HS	65.45 ± 0.46	This work
HS HSA Free	BLQ	This work
CSF	BLQ	This work

CAMHB: iron depleted cation adjusted Mueller Hinton, HSA: human serum albumin, HPF: human pleural fluids, HS: human serum, CSF: cerebrospinal fluid, BLQ: below limit of quantification.

[1] C. Pimentel *et al.*, "Human pleural fluid and human serum albumin modulate the behavior of a hypervirulent and multidrug-resistant (MDR) acinetobacter Baumannii representative strain," *Pathogens*, vol. 10, no. 4, pp. 1–13, 2021, doi: 10.3390/pathogens10040471.

Figure S1. SDS-PAGE of human fluids analyzed using the ProteoExtract® Albumin/IgG Removal Kit (Sigma-Aldrich, MA, United States)

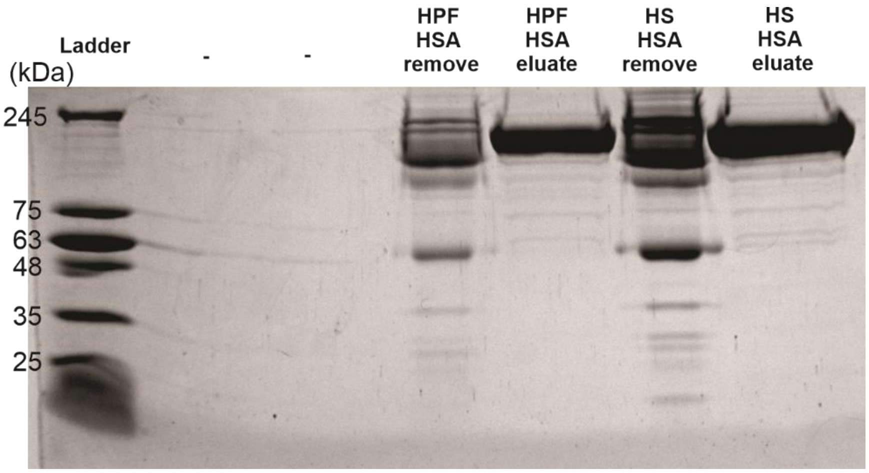

HSA: human serum albumin, HPF: human pleural fluids, HS: human serum.
